# Outcomes of and barriers to cataract surgery in Sao Paulo State, Brazil

**DOI:** 10.1186/s12886-017-0637-6

**Published:** 2017-12-22

**Authors:** Gabriel de Almeida Ferreira, Luisa Fioravanti Schaal, Marcela Dadamos Ferro, Antonio Carlos Lottelli Rodrigues, Rajiv Khandekar, Silvana Artioli Schellini

**Affiliations:** Universidade Estadual Paulista Julio de Mesquita Filho Faculdade de Medicina Campus de Botucatu Botucatu, Sao Paulo, Brazil

**Keywords:** Cataract, Blindness, Treatment Outcome, Health services accessibility

## Abstract

**Background:**

Cataract is the leading cause of blindness in developing countries and identification of the barriers to accessing treatment is essential for developing appropriate public healthcare interventions. To evaluate the barriers to cataract surgery after diagnosis and assess the postoperative outcomes in Sao Paolo State, Brazil.

**Methods:**

This prospective study evaluated cataract patients from 13 counties in São Paulo State in 2014. Cataract was diagnosed in the community by a mobile ophthalmic unit and patients were referred to a hospital for management. Gender, age, distance to the hospital and local municipal health structure were evaluated as possible barriers. Data were analyzed for postoperative outcomes and the impact on blindness and visual impairment.

**Results:**

Six hundred patients were diagnosed with cataract with a mean age of 68.8±10.3 years and 374 (62.3%) were females. Two hundred and fifty-four (42.3%) patients presented to the referral hospital. One hundred forty-four (56.7%) underwent surgery, 56 (22.0%) decided not to undergo surgery, 40 (15.7%) required only YAG-Laser and 14 (5.5%) required a spectacle prescription only. Visual acuity increased statistically significantly from 1.07±0.73 logMAR at presentation to 0.25±0.41 logMAR at the final visit after intraocular lens implantation (*p*=0.000). There was a statistically significantly decrease from 17 (11.8%) blind patients and 55 (38.2%) visually impaired patients at presentation to 2 (1.4%) and 5 (3.5%) patients respectively after treatment (*p*=0.000).

**Conclusion:**

Less than half of the individuals with cataract presented to the hospital for surgery. Among the patients who underwent treatment, there was an overall decrease in the number of blind individuals and visually impaired individuals. The barriers to cataract surgery were older age, greater distance to the hospital, municipalities with fewer inhabitants and less ophthalmic services.

## Background

Cataract surgery is a leading cause of blindness in developing countries [1]. In 2010, there were an estimated 39 million blind individuals and 285 million visually impaired individuals globally. Cataract was considered the primary cause of blindness, responsible for 51% of these cases [2]. In 1990, the World Health Organization (WHO) created “Vision 2020 – the right for sight” initiative, which aims to eliminate avoidable blindness by 2020 worldwide. Despite improvement in access to surgery, many regions worldwide do have adequate coverage for cataract surgery [3].

Barriers to access cataract surgery differ by regions and include, gender, fear of surgery, status of visual disability, educational level, visual needs, distance from the care provider, cost and lack of an escort/caretaker [4].

Brazil has a universal public health system (*Sistema Único de Saúde – SUS)* that provides surgery without cost to those in need [5]. Despite universal healthcare, cataract remains the major cause of blindness in Brazil [6, 7]. Appropriate public healthcare strategies can be developed to eliminate cataract as a source of blindness using data from studies of barriers to cataract surgery in regions of Brazil. Currently there are no published studies of barriers to cataract surgery in a Brazilian population. The present study evaluates the barriers to cataract surgery and presents some suggestions to increase the uptake of cataract surgery.

## Methods

A cross-sectional prospective survey was performed in the southwest region of São Paulo State, Brazil, involving patients who were screened at a Ophthalmic Mobile Unit (OMU) in 2014. SUS covered all the costs for treatment. Patients were screened in 13 municipalities (Table 1). The tertiary health reference center for the 13 municipalities was the Clinical Hospital of Botucatu Medical School (*Hospital das Clínicas da Faculdade de Medicina de Botucatu –* HCFMB*)*. This study was approved by the Ethics Committee of the Faculdade de Medicina de Botucatu – UNESP, Sao Paulo, Brazil and adhered to the tenets of the Declaration of Helsinki. All study subjects signed a consent form.

**Table 1:** Characteristics of the municipalities served by the Ophthalmic Mobile Unit in 2014

	Total patient referred n (%)	Inhabitants n	Per capita income (R$)	M-HDI	Distance to the Hospital (km)	Number of ophthalmologists n
Agudos	42 (7.0)	34524	627.75	0.745	75.3	^a^
Barra Bonita	99 (16.5)	35246	1056.38	0.788	60.0	4
Boracéia	41 (6.8)	4268	708.05	0.754	106.0	^a^
Brotas	57 (9.5)	21580	807.88	0.817	92.3	1
Conchas	27 (4.5)	16288	830.35	0.736	56.8	1
Dois Córregos	13 (2.2)	24761	896.77	0.725	81.3	1
Iacanga	30 (5.0)	10013	758.67	0.779	150.2	2
Igaraçu do Tietê	81 (13.5)	23362	650.03	0.727	53.3	1
Macatuba	50 (8.3)	16259	1065.29	0.777	68.8	1
Óleo	19 (3.2)	2673	788.27	0.761	133.3	2
Piramboia	19 (3.2)	5653	549.16	0.681	44.2	^a^
Promissão	102 (17.0)	35674	714.65	0.724	217.0	^a^
Taquarituba	20 (3.3)	23163	721.89	0.741	137.4	1

^a^Information not provided by municipalities

n=number

The UMO team was composed of ophthalmologists and local health workers from each municipality. Subjects underwent a comprehensive ocular exam. Visual acuity (VA) was evaluated using an illuminated Snellen E chart and the values were converted to the logarithm of the minimum angle of resolution (logMAR) for statistical analysis. The Snellen to logMAR conversion was as follows: counting fingers, hand movement, light perception and without light perception corresponded to 2.10, 2.40, 2.70 and 3.00, respectively [8]. The WHO definitions were used to classify vision as follows: blindness was defined as VA<20/400 and visual impairment was 20/400<VA<20/60 in the better eye with the best optical correction [9].

All participants underwent an objective and subjective refraction with an autorefractor (Accuref-K; Shinn Nippon, Tokyo, Japan) and a manual refractor (RT 6000; Nidek Co. Ltd., Gamagori, Japan). Slit lamp biomicroscopy (Shinn Nippon, Tokyo, Japan) was performed to evaluate the anterior segment and the posterior segment using a 90 D Volk lens. For patients who did not achieve good vision with refraction, a dilated examination was performed (Mydriacyl; Alcon Inc., Fort Worth, TX, USA) for comprehensive evaluation of the lens and fundus. Intraocular pressure (IOP) was measured with air-puff tonometer (CT-60; Topcon Corp., Tokyo, Japan). For patients with IOP over 20 mmHg, Goldmann tonometry was performed to confirm the air-puff tonometry readings (Haag-Streit AG, Köniz, Switzerland).

To ensure consistency, survey staff were trained, periodically monitored and the equipment was calibrated regularly. All data collection sheets were pretested.

After the ophthalmic exam, patients diagnosed with cataract or pseudophakia with posterior capsule opacification (PCO) were referred to the HCFMB for further examination, YAG (yttrium aluminum garnet) laser or surgery and an appointment was scheduled. The municipalities provided transportation to the hospital on the day of the appointment.

At the hospital, the patient underwent another ophthalmic examination. Biometry was performed using the IOLMaster 500 (Carl Zeiss Meditec, Jena, Germany) and IOL calculations targeted emmetropia in all eyes. In eyes with dense cataracts that precluded optical biometry, the axial length was measured with an ultrasonic biometer (SP-1000AP; Sonoptek, Beijing, China) and the IOL power was calculated with the IOLMaster.

Patients underwent phacoemulsification or extracapsular cataract extraction (ECCE) based on surgeon preference. All patients underwent IOL implantation.

### Statistical analysis

To analyze demographics and outcomes after treatment, the data obtained at the OMU visit were transferred to an Excel spreadsheet (Microsoft Corp., Redmond, WA, USA). The electronic medical records from the hospital were used to collect data on the diagnosis, surgical procedure and postoperative outcome and transferred to an Excel spreadsheet.

National data were consulted to determine possible barriers for evaluation in this study relating to patient adherence to the proposed treatment. The socioeconomic and demographic data of the assisted municipalities, such as Human Development Index (HDI), *per capita* income and number of inhabitants, were obtained from the *Instituto Brasileiro de Geografia e Estatística* – 2010 (IBGE) [10]. Data on the infrastructure of the ophthalmic service of the participating municipalities were collected using a standardized questionnaire, answered by the official representative of Public Health Care for the municipality.

Data analysis was performed with SPSS 22.0 software (IBM Corp., Armonk, NY, USA). The frequency, mean and standard deviation were calculated. Normally distributed data were analyzed by the Kolmogorov-Smirnov and Shapiro-Wilk tests. Statistical significance was indicated by *p*<0.05.

## Results

During the study period, 600 patients from 13 participating municipalities were diagnosed with cataract or PCO and referred to the HCFMB. The mean age of the patients was 68.8 ± 10.3 years, of which 374 (62.3%) were female, 46 (7.7%) were blind and 202 (33.7%) were visually impaired (Table 2).

**Table 2:** General characteristics of the six hundred individuals referred to the reference hospital in 2014

Age (years)	68.8±10.3^a^
Best Corrected Visual Acuity (logMAR)	0.60±0.53^a^
Gender	
Female	374 (62.3)
Male	226 (37.7)
Blindness	46 (7.7)
Visual Impairment	202 (33.7)
Attended to the reference center	254 (42.3)

^a^mean± standard deviation n (%)

**Fig. 1:**
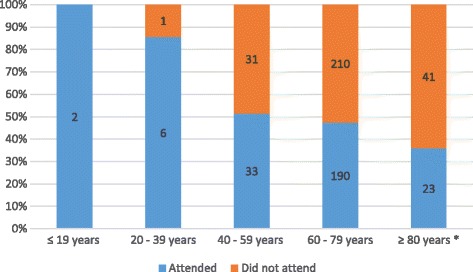
Comparison between age groups who attended or did not attend the scheduled visit to the referral hospital

Two hundred and fifty-four (42.3%) patients presented for scheduled care. Presentation to the referral hospital varied between 16% to 63% among the municipalities. Younger patients had a statistically greater tendency to present to the referral hospital (67.4±11.3 years vs. 70.0±9.1 years, *p*=0.004) (Fig. 1), with no statistical influence of gender (*p*>0.05). Attendance was statistically associated with visual impairment (*p*=0.000) but not blindness (*p*>0.05) (Table 3).

**Table 3:** Comparison of individuals who attended or did not present to the reference hospital in 2014

	Attended (*n*=254) - *n*(%)	Did not present (*n*=346) - *n* (%)	*p*
Age (years)	67.4±11.3^a^	70.0±9.1	**0.004**
Best Visual Acuity (logMAR)	0.62±0.55^a^	0.59±0.52	0.631
Gender			
Female	155 (61.0)	219 (63.3)	0.571
Male	99 (39.0)	127 (36.7)	
Blindness	24 (9.4)	22 (6.4)	0.160
Visual Impairment	106 (41.7)	96 (27.7)	**<0.001**

^a^mean ± standard deviation

n=number; *p<0.05* is statistically significant (bold values)

Of the 254 patients who attended the hospital, 144 (56.7%) underwent surgery in at least one eye, 56 (22.0%) did not undergo surgery due other ophthalmic comorbidities or because the patient refused surgery, 40 (15.7%) were pseudophakic with PCO that required YAG capsulotomy, and 14 (5.5%) had milder lens opacities and reasonable VA with spectacle correction and remained under observation.

YAG capsulotomy was performed in 65 eyes of 40 patients, resulting in a statistically significant improvement in VA from 0.93±0.73 logMAR before capsulotomy vs 0.25±0.46 logMAR after capsulotomy, *p*<0.000). Prior to YAG laser treatment there were four (10.0%) blind patients and 11 (27.5%) visually impaired patients. After YAG capsulotomy there was a statistically significant reduction in the number of blind patients to zero and visually impaired patients to 2 (2.0%) (*p*<0.000, both comparisons).

Of the 56 patients who did not undergo surgery, 27 (48.2% of those who did not undergo surgery and 10.7% of all patients) had surgery postponed due to mild lens opacity, 17 (30.4) were lost to follow-up before surgery and 9 (16.1%) refused surgery.

A total of 253 surgeries were performed, of which 245 were phacoemulsification and 8 were extracapsular cataract extraction (ECCE), all with IOL implantation. There was a statistically significant increase in VA from 1.07±0.73 logMAR (20/225 Snellen acuity) preoperatively to 0.25±0.41 logMAR (20/32 Snellen acuity) at last postoperative visit (*p*=0.000). The mean improvement is VA was -0.86 logMAR, which is equivalent to 8 lines on an early treatment of diabetic retinopathy study (ETDRS) chart.

Of the patients who underwent surgery, 37 (14.6%) had an associated ocular comorbidity and, in 32 (12.6%) of these patients the comorbidity caused the low VA. The most common comorbidity was, age-related macular degeneration (AMD) in 11 (33.4%) cases. Comparison of groups with and without comorbidities justifying the low VA indicated, statistically significant differences in the preoperative VA (1.40±0.80 logMAR vs 0.83±0.70 logMAR; *p*=0.000) and the postoperative VA (1.05±0.82 logMAR vs 0.21±0.40 logMAR; *p*=0.000).

Preoperatively 17 (11.8%) patients who underwent phacoemulsification in at least one eye were classified as blind and 55 (38.2%) were considered visually impaired. There was a statistically significant decrease after phacoemulsification to 2 (1.4%) blind patients and 5 (3.5%) patients who were visually impairment (*p*=0.000). Of those who remained blind or visually impaired after phacoemulsification, 4 (57.1%) the low VA could be explained by an ocular comorbidity.

The final postoperative refraction was available for 154 (60.9%) eyes. At the final postoperative visit the mean spherical was -0.32 ± 1.13 D (range, -3.75 D to +2.75 D), the mean cylindrical was -1.02 ± 1.02 D (range -9.00 D to 0.00 D) and the mean spherical equivalent was -0.83±1.11 D (range -3.88±2.13).

There were 209 patients who underwent cataract surgery and YAG capsulotomy, of whom 21 (10.0%) were blind and 16 (7.6%) were visually impaired prior to treatment. After treatment, there was a statistically significant decrease in the number of blind patients to 2 (0.9%) and 7 (3.3%) remained visually impaired (*p*<0.000).

Data on local demographics and municipal health services was provided by 9 of the 13 municipalities covered in the study. Hence data on 396 patients could be analyzed for attendance and determination of possible barriers (Table 4).

**Table 4:** Demographic data of 396 individuals and the municipalities of origin based on attendance in 2014

	Attended (*n*=168); (%)	Did not attend (*n*=228); n (%)	*p*
Age (years)	69.3±9.2^a^	67.3±12.0^a^	0.081
Best Visual Acuity (logMAR)	101 (60.1)	151 (66.2)	0.245
Gender			
Female	67 (39.9)	77 (33.8)	
Male	9 (5.4)	21 (9.2)	0.181
Blindness	46 (27.4)	59 (25.9)	0.818
HDI	0.763±0.034	0.770±0.029	**0.034**
Distance to the specialized hospital (km)	73.6±28.9	83.2±32.9	**0.000**
Per capita income (R$)	836.6±164.1	890.6±156.1	**0.000**
Inhabitants	24287.9±8349.1	21923.7±9642.4	**0.000**
Patients with blindness prevention program	8 (4.8)	42 (18.4)	**0.000**
Patients with ophthalmologist in county of origin	155 (92.3)	192 (84.2)	**0.020**
Hospital with structure for facectomy	16 (9.5)	54 (23.7)	**0.000**
Ophthalmic Apparatus in the basic health	66 (39.3)	116 (50.9)	**0.025**
Cataract campaign in the last five years	154 (91.7)	209 (91.7)	1.000
Total	168 (%)	228 (%)	

^a^mean±standard deviation

n=number; *p*<0.05 is statistically significant (bold values)

For patients who did not present for their scheduled visits, the municipal HDI was statistically significantly higher (0.772±0.029 versus 0.763±0.034, *p*=0.034), there was a statistically greater distance to the hospital (83.2±32.9 km vs 73.6±28.9 km, *p*=0.00), a statistically higher per capita income (R$890.6±156.1 vs R$836.6±164.1, *p*=0.000) and statistically lower population (21,923.7±9,642.4 inhabitants vs 24,287.9± 8,349.1 inhabitants, *p*=0.000).

Regarding the municipality health structure, 84.2% of patients who did not present for the scheduled visit had ophthalmologists in their municipality of origin, compared to 92.3% of those who attended (*p*=0.016). Additionally, patients who did not attend had statistically significantly more blindness prevention programs (18.4% vs 4.8%, *p*=0.000), statistically better local coverage by ophthalmic surgeons (18.4% vs 4.8%, *p*=0.000), statistically greater availability of ophthalmic equipment at municipal primary healthcare facilities (50.9% vs 39.3%, *p*=0.022) and statistically better municipal hospital services and infrastructure to perform phacoemulsification (23.7% vs 9.5%, *p*=0.000).

## Discussion

The outcomes of this cross-sectional study of cataract patients indicated that those who presented to the referral hospital were younger and mainly females. This observation is similar to other Brazilian studies [11–13] and is likely due to the characteristics of the Brazilian population which has a greater number of elderly women (IBGE – 2010) [10]. Although the current study reported greater female presentation to the referral hospital, there was no statistical difference between genders (*p*>0.05).

In the current study, a relatively low number of patients (42.3%) diagnosed with cataract (based on the OMU screening) presented to the specialized hospital for phacoemulsification or YAG capsulotomy.

In the entire study sample, 10.0% of patients were blind and 7.6% were visually impaired at presentation. After treatment, there was a statistically significant decrease in blindness to 0.9% of patients and 3.3% of the patients were visually impaired (*p*<0.000). Hence, despite the low patient presentation rate for referral, the treatment was effective and achieved the WHO criteria. The WHO recommends a maximum of 5.0% of patients with best corrected visual acuity less than 20/400 (blindness) after cataract surgery.

A British study of 127,658 patients reported an improvement in VA from 0.63 logMAR at baseline to 0.16±0.30 logMAR after cataract surgery [8]. The VA outcomes of the current study are lower than the British study [8]. The differences in outcomes between studies are like because the British study was performed in a developed country, on a larger sample size and with earlier diagnosis. In the current study, many patients had associated ocular comorbidity that resulted in the low VA at final visit. However, the postoperative VA in patients without comorbidities in the current study was similar to the British study [8]. A recent study from São Paulo, Brazil reported a decrease from 17.6% blind patients and 23.5% visually impaired at baseline, to 5.9% and 11.8% respectively, after cataract surgery [14]. The VA outcomes from the current study are well within or exceed the range reported from other developing countries. For example, a study of cataract surgery in a rural province in Laos reported 9.5% blind and 44.3% visually impaired postoperatively [15]. In Nigeria, 58.5% remained blind and 16.1% remained visually impaired after surgery [16]. In Nepal, 8.0% of patients remained blind and 22.0% remained visually impaired after surgery [17]. In Pakistan, 36.8% remained blind and 22.0% remained visually impaired [18]. The poorer VA outcomes at final postoperative visit from some of the other developing countries are likely due to the uncertain surgical conditions, often treating cases where biometry was difficult to perform, or patients were aphakic. Additionally, in some of the other developing countries standard cataract surgery techniques may not be possible the surgeons may need to improvise based on the surgical environment [19, 20]. However, in the current study cataract surgery was performed in a specialized tertiary hospital with good infrastructure with the option of IOL implantation in all patients which explains the good postoperative outcomes.

The number of surgeries, the quality of surgery and the final VA are all factors for achieving the goal of Vision 2020. Access to healthcare services is another barrier despite the strategy of approaching patients in their hometown using an OMU and detecting individuals who require surgery. For example, despite these initiatives, only 42.3% of the screened patients presented to the specialized hospital in the current study.

Our analysis of the social, economic and demographic characteristics and the health structure of the municipalities indicated that greater distance from the hospital, with higher HDI, higher per capita income and a lower municipal population were most unlikely to present to the referral hospital. These characteristics can be considered the barriers to treatment of cataracts in Sao Paolo State, Brazil. Interestingly the same barriers were reported in a rural region of China [21] and in central Ethiopia [22]. The factors “higher per capita income” and “higher HDI” differ from other studies, which described insufficient family income and an underdeveloped population as important barriers [21–23]. Perhaps patients with higher per capita income are able to undergo treatment in their hometown or elsewhere, bearing the expenses of the procedure. This observation may explain the lack of presentation to the referral hospital. Other factors that contribute to the low presentation rate for ophthalmic surgeries are, comorbidities, fear of the operation or of becoming blind postoperatively [4, 14].

There are an average 62 ophthalmologists per 1 million inhabitants in Latin America and this number is increasing. Therefore, there are an adequate number of ophthalmologists for coverage of cataract surgery [24]. However, the number of ophthalmologists that perform cataract surgery and how many surgeries each ophthalmologist performs remains unknown. Unlike a previous study [24], we found that the majority of patients who did not present to the referral hospital had a higher number of ophthalmologists in their hometown. Despite the unfavorable presentation, patients who did not attend can have their cataracts addressed in their own municipalities. These municipalities may have an adequate number of ophthalmologists, surgeons and hospital infrastructure for cataract surgery.

There are some limitations to the present study, including the lack of data on the best-corrected VA for all patients and that the barriers were not analyzed individually. Separate analyses of the barriers were not performed because we collected generalized data regarding the study population. However, there is a relative paucity of data from studies evaluating a Brazilian sample with an OMU. Hence, the outcomes of the current study provide data that can be used to allocate adequate resources and develop public healthcare initiatives.

The outcomes of the current study indicate that the elimination of cataract as a cause of blindness and/or visual impairment in Brazil requires greater coordination between the municipality and the regional tertiary hospital to ensure greater uptake of surgery.

## Conclusion

Less than half of the patients diagnosed with cataract in municipalities using an OMU actually presented to a specialized hospital for treatment despite referral. However, the outcome of phacoemulsification was encouraging, resulting in a significant reduction of blind and visually impaired patients.

The main barrier to attendance were advanced age, greater distance to the specialized hospital and municipalities with lower populations. However, the presence of blindness prevention programs, ophthalmic surgeons, available ophthalmic equipment at healthcare centers and hospital with the resources to perform phacoemulsification may be factors that reduce adherence to appointments at a specialized hospital.

## Abbreviations

AMD: Age-related macular degeneration
ECCE: Extracapsular cataract extraction
ETDRS: Early treatment of diabetic retinopathy study
HCFMB: Hospital das Clínicas da Faculdade de Medicina de Botucatu
HDI: Human Development Index
IBGE: Instituto Brasileiro de Geografia e Estatística
IOL: Intraocular lens
IOP: Intraocular pressure
logMAR: logarithm of the minimum angle of resolution
OMU: Ophthalmic Mobile Unit
PCO: Posterior capsule opacification
SUS: Sistema Único de Saúde
VA: Visual acuity
WHO: World Health Organization
YAG: Yttrium aluminum garnet
